# Pharmacodynamic Evaluation and Mechanism of Ginseng Polysaccharide against Nephrotoxicity Induced by Hexavalent Chromium

**DOI:** 10.3390/nu16101416

**Published:** 2024-05-08

**Authors:** Baitong Jing, Mengyao Wei, Huaguo Chen, Wen Xie, Silan An, Jiawen Li, Shenglin Wang, Xin Zhou

**Affiliations:** 1 Key Laboratory for Information System of Mountainous Areas and Protection of Ecological Environment, Guizhou Normal University, 116 Baoshan North Rd., Guiyang 550001, China; 18690724636@163.com (B.J.); j13634531136@163.com (M.W.); chenhuaguo1981@163.com (H.C.); 18846437313@163.com (W.X.); 15121506267@163.com (S.A.); 13555316428@163.com (J.L.); m15085807646@163.com (S.W.); 2 Guizhou Engineering Laboratory for Quality Control & Evaluation Technology of Medicine, Guizhou Normal University, 116 Baoshan North Rd., Guiyang 550001, China; 3 The Research Center for Quality Control of Natural Medicine, Guizhou Normal University, 116 Baoshan North Rd., Guiyang 550001, China

**Keywords:** ginseng polysaccharide, hexavalent chromium, mechanism of action, lipidomics

## Abstract

Hexavalent chromium is a common pollutant in the environment. Long-term exposure to hexavalent chromium can cause damage to multiple organs. The kidney is one of the main organs that metabolizes heavy metal toxicity, and the accumulation of Cr (VI) in the body can lead to serious damage to kidney function. Studies have shown that ginseng polysaccharides have the function of preventing cisplatin-induced endoplasmic reticulum stress, inflammatory response, and apoptosis in renal cells, but their efficacy and mechanisms against hexavalent chromium-induced nephrotoxicity need to be explored. The aim of this study was to explore the efficacy and mechanism of ginseng polysaccharide against hexavalent chromium-induced nephrotoxicity. The results of pharmacodynamic experiments showed that ginseng polysaccharide could significantly reduce the kidney index, urea nitrogen (BUN), and serum creatinine (Cre) values of K_2_Cr_2_O_7_-treated mice. The results of mechanistic experiments showed that ginseng polysaccharides could alleviate oxidative stress, apoptosis, and biofilm damage in renal tissues caused by Cr (VI). Lipidomic correlation analysis showed that ginseng polysaccharides could protect the organism by regulating the expression of differential lipids. This study opens new avenues for the development of alternative strategies for the prevention of kidney injury caused by hexavalent chromium.

## 1. Introduction

Chromium is a heavy metal and the 21st element in the Earth’s crust [1]. Excessive chromium intake can cause multiple tissue and organ damage in the body. The toxicity of different valence chromiums is different. A large number of data points show that Cr (II) is easy to oxidize and does not exist in the biological body. Therefore, Cr (II) and chromium metal itself have limited or no toxicity, while Cr (III) is the most stable oxidation state and the most common in living organisms. Cr (III) is not easily absorbed in the gastrointestinal tract and combines with proteins on the skin surface to form a stable complex; it also does not cause dermatitis or skin ulcers, so it has minor toxicity [2]. Cr (VI) was the most toxic. Based on the available scientific evidence, the International Agency for Research on Cancer (IARC) has classified hexavalent chromium as a Group 1 carcinogen and has recognized that Cr (VI) is highly toxic and has been identified as one of the most significant pollutants in the environment and in living organisms [3,4]. Cr (VI) enters the body mainly through dermal contact, inhalation of the respiratory tract, and ingestion of the digestive tract, which are three ways to enter the body that may cause significant damage [5]. Cr (VI) can easily cross the cell membrane and enter the cell. After being reduced by reducing agents in the cell, Cr (III) with low toxicity is accumulated in the liver, kidney, and other organs [6].

The mechanisms of renal injury leading to chromium toxicity are multifaceted. Studies have shown that after Cr (VI) enters the cell, intracellular reductants (e.g., ascorbic acid, malondialdehyde, etc.) reduce it to Cr (III), which enters and accumulates in the cell in the form of chromate by means of non-specific channels in the cell membrane. Excessive ROS (reactive oxygen species) are generated during the redox reaction, which leads to toxicity [7,8,9,10,11,12]. Chromium toxicity-induced ROS production leads to cellular dysfunction, apoptosis, and death. Zheng studies have shown that Cr (VI) induces the disorder of mitochondrial dynamics in rat kidneys via inhibition of the silent information regulator two ortholog 1/peroxisome proliferation-activated receptor-g coactivator-1a pathway [13]. Hexavalent chromium exposure led to renal dysfunction through oxidative injury and alterations in the antioxidant defense system in addition to histopathological and immunohistochemical changes [14]. Cr (VI)-induced renal dysfunction was clearly identified through urine protein drastically, and the serum creatinine and blood urea nitrogen levels were augmented [15].

According to some existing studies, chemicals that have protective effects on Cr (VI)-induced kidney damage can be roughly divided into two categories: Metal chelators and antioxidants [16]. Chelating agents reduce toxicity by calculating Cr (VI), and some better-known examples include ethylenediamine tetraacetic acid (EDTA), diethlenetriaminepentaacetic acid (DTPA), dimercaptosuccinic acid (DMSA), and dimercaptopropanesulfonic acid (DMPS). Antioxidants relieve chromium poisoning by eliminating free radicals in the body, typically including endogenous glutathione (GSH) and anti-bad blood acid (VC) [17]. However, metal chelating agents also have disadvantages, such as higher prices, side effects, and being difficult to obtain. Therefore, the search for low side effects and easily accessible drugs to protect against hexavalent chromium-induced nephrotoxicity is of great practical importance.

Numerous studies in recent years have shown that plant polysaccharides can effectively relieve various forms of kidney damage, which has made them widely used in treating kidney disease [18]. Ginseng is a traditional medicinal plant in China, and its use in traditional Chinese medicine dates back approximately 5000 years. It has therapeutic properties, such as tonifying the vital energy, regulating the five viscera, and dispelling evil spirits. Polysaccharides have likewise attracted academic attention as important active ingredients. Ginseng polysaccharides with different structural characteristics and activities have been gradually emphasized as volatile and active components of ginseng in traditional Chinese medicine [19]. Studies have shown that ginseng polysaccharide supplementation can improve growth immunity and alleviate liver function abnormalities, among other effects [20]. El-Mahalaway’s study showed that Ginseng has a protective effect against potassium dichromate-induced nephrotoxicity, as it has antioxidant, anti-inflammatory, and antiapoptotic activities [21]; however, the pharmacodynamic evaluation and mechanisms of ginseng polysaccharide on renal injury induced by hexavalent chromium are still unclear.

Pharmacodynamic studies can provide a lot of parameter information for drug treatment, such as appropriate drug concentration, measurable drug duration, and drug activity [22]. Liu’s research showed that, after cadmium-induced renal injury, the efficacy of Opuntia dillenii polysaccharide reached its highest level within 7 days of treatment, and the trend continued to 35 days after 14 days of continuous administration. Different doses of ODP showed a good dose−effect relationship [23]. We conducted a preliminary experiment and found that ginseng polysaccharides had a certain mitigation effect on renal injury caused by hexavalent chromium; however, there was no in-depth study on the efficacy because we conducted pharmacodynamic evaluation experiments in regard to both the time-effect and dose-effect. 

In this study, a renal injury model was established using potassium dichromate (K_2_Cr_2_O_7_), and different doses of ginseng polysaccharide (25/50/100/200/400 mg∙kg^−1^) were administered to hexavalent-chromium-poisoned mice. Ginseng polysaccharide was administered at different doses (25/50/100/200/400 mg∙kg^−1^) to hexavalent-chromium-induced nephrotoxicity mice to investigate the time-effect and dose-effect relationships. Additionally, the effectiveness of the drug was evaluated based on general symptoms in mice, kidney index, renal function indicators (serum myeloid, Cre, hemourinic nitrogen, BUN), and renal tissue pathology observations. Finally, based on the two aspects of traditional target and non-target liposuction, the toxicity of GPS anti-Cr (VI) was studied, and oxidative stress markers (SOD, MDA, GSH), cell death markers (Casp-3, Bcl-2/Bax), cell membrane damage markers (ATPase, Ca^2+^-ATPase), and the histological approach of a non-targeted liquid−liquid coupling technique were used to resolve the differential expression of lipids between different groups of mice GPS anti the mechanism of Cr (VI)-induced nephrotoxicity was investigated at the molecular level. It is hoped that this study will provide useful information for the development of ginseng-based nutritional products and adjuvant drugs. 

## 2. Materials and Methods

### 2.1. Materials

Polysaccharides extracted from the rhizome of Changbai Mountain Ginseng were purchased from the Baichuan KangZe Biology Science and Technology Co., Ltd (Xi’an, Shaanxi, China). The purity of GPS is greater than or equal to 90%. Potassium dichromate, formaldehyde, Xylene, n-Butanol, methanol, acetonitrile, and isopropyl alcohol were purchased from Chongqing Guangdong Chemical Co., Ltd. (Chongqing, China). Dimercaptosuccinic acid was purchased from Shanghai Xinya Pharmaceutical Minhang Co., Ltd. (Shanghai, China). Mouse BUN, CRE, SOD, GSH, MDA, Casp-3, Bcl-2, Bax, ATPase, and Ca^2+^-ATPase reagent boxes were purchased from Shanghai Qiaoyu Biotechnology Co., Ltd. (Shanghai, China). Physiological salt water was purchased from Guangzhou Cologne Pharmaceuticals Co., Ltd. (Guangzhou, China). Water-free ethanol was purchased from Tianjin Zhiyuan Chemical Reagent Co., Ltd. (Tianjin, China). Neutral red stain was purchased from Beijing Solebro Technology Co., Ltd. (Beijing, China). Hematoxylin-Eosin staining solution was purchased from Sinopharm Chemical Reagent Co., Ltd. (Beijing, China). Methyl tert-butyl ether was purchased from Guiyang Ron Chemical Reagent (Guiyang, Guizhou, China). Centrifugal tubes were purchased from Beijing Lanjie Technology Co., Ltd. (Beijing, China).

### 2.2. Animals and Experimental Design

This study used male ICR mice (4–6 weeks old, 18–22 g). Changsha Tianqin Biotechnology Co., Ltd. (Changsha, Hunan, China, license number: SCXK (X) 2019-0013) provided the animals. All mice were kept in a temperature-controlled environment at 25 ± 2 °C with a relative humidity of 60 ± 10%. The mice were provided with a standard laboratory diet and water, and they were cared for and treated in accordance with the China Animal Protection Commission’s requirements, which was authorized by the Guizhou Normal University Animal Care and Use Commission.

After 5 days of adaptive feeding, a pharmacological evaluation of the nephrotoxicity caused by GPS anti-Cr (VI) was first made from the time-effectiveness point of view. The 200 mice were randomized into five groups at random, each with four groups (*n* = 10): the Normal Control Group (NC), the Model Control Group (MC), the Positive Control Group (PC), and the Ginseng polysaccharide treatment group (GPS), for a total of 20 groups. The experiment was performed according to the procedure shown in Table 1. Based on previous studies, the dose of K_2_Cr_2_O_7_ used in kidney injury modeling was 50 mg·kg^−1^ [24]. A group of mice was killed each week to evaluate kidney function. The optimal time of administration was determined by comparing the proximity of kidney function between each GPS group and the NC group. When the trial was extended to the fifth week, a decision was made on whether to continue treatment based on changes in kidney function in the first four groups of mice. If the condition was stable, treatment was stopped in the fifth batch to evaluate kidney function; otherwise, the rest of the patients would continue to receive treatment.

The pharmacodynamic evaluation of GPS against Cr (VI)-induced nephrotoxicity was designed from the perspective of dose efficacy. A total of 80 ICR mice were adapted to feed for 5 days. The mice were randomly divided into eight groups (*n* = 10): the normal control group (NC), the model-control group (MC), the positive controlling group (PC), and the polysaccharide treatment group (GPS1, 2, 3, 4, 5). The experimental process is shown in Table 2. The mice were executed at the end of the experiment and the renal functions were assessed. The optimal dose was determined by comparing the degree of renal function recovery in different GPS groups.

### 2.3. Collection of Blood and Kidney Tissue

The mice were anesthetized and orbital blood collection was performed in 1.5 mL wash sterile centrifuge tubes, left at room temperature for 30 min, then centrifuged (3000 r·min^−1^) to separate the serum from the red blood cells. The serum was then collected and stored in a −20 °C refrigerator for later use. The mice were killed via cervical dislocation, and their kidneys were rapidly shaved and removed on both sides. The kidneys were cleansed with normal saline, and the surface moisture was dried with filter paper before being weighed with an electronic scale. The mice’s ipsilateral kidneys were immediately submerged in a formalin solution for further usage.

### 2.4. Pharmacodynamic Study of Ginseng Polysaccharide on Nephrotoxicity Induced by Hexavalent Chromium in Mice

#### 2.4.1. Monitoring of General Signs in Mice

Feed consumption, coat color, diet, mental state and metabolism of the mice were observed and recorded daily. The mice were weighed every 3 days, and feed and drinking water were provided as usual until after the last administration, when the mice were fasted for 12 h and their final weights were weighed.

#### 2.4.2. Measurement of Kidney Index

After weighing the kidney, the following formula was used:

$$kidney\;index=ipsilateral\;kidney\;mass\left(g\right)/mouse\;body\;weight\left(g\right)\times 100\%$$

#### 2.4.3. Detection of Renal Function Biomarkers

The serum was thawed at 4 °C, and the sample, standard, antibody, and color rendering agent were added according to the kit instructions. The absorbance (OD value) was measured at a 450 nm wavelength with a microplate reader to determine the serum biochemical indicators, blood urea nitrogen (BUN), and serum creatinine (CRE).

#### 2.4.4. Renal Histopathological Examination

The kidney tissue was removed from the formalin solution and washed with water for 10 min. Each group was cut into miniature pieces of square tissue, numbered, and placed in an embedding box. The KH-TS automatic dehydrator was used for dehydration treatment. The kidney tissue of each group of mice after dehydration was entirely immersed in liquid paraffin. Paraffin blocks containing kidney tissue were prepared using an embedding mould and the KH-BQ automated embedding machine. Kidney samples were stained with hematoxylin-eosin (ethanol), and 5 micron sections were cut and photographed with an MF-3 inverted fluorescence microscope for histopathological observation.

### 2.5. Study of the Mechanism of Ginseng Polysaccharide in Nephrotoxicity Induced by Hexavalent Chromium in Mice

#### 2.5.1. Detection of Oxidative Stress Markers in Renal Tissue

Place 0.1 g of thawed kidney tissue into a 5 mL centrifuge tube, add an appropriate amount of normal saline, and mash it with a homogenizer. After centrifugation at 3000 r·min^−1^ for 10 min at 4 °C, the supernatant was collected. The expression levels of SOD, MDA, and GSH were detected by ELISA. ELISA kits were purchased from Shanghai Qiaoyu Biotechnology Co., Ltd. (Shanghai, China). According to the kit instructions, samples, standards, antibodies, and chromogenic agents were added in turn. A microporous reader was used to detect the absorbance (OD value) at the 450 nm wavelength, and the amounts of SOD, MDA, and GSH in mouse serum were then determined.

#### 2.5.2. Detection of Apoptosis Biomarkers

The appropriate amount of renal tissue homogenate was taken and operated on strictly according to the kit instructions (consistent with the Section 2.5.1 operation steps) to detect the levels of Casp-3, Bcl-2, and Bax in the kidney.

#### 2.5.3. Detection of Cell Membrane Damage Index

An appropriate amount of renal tissue homogenate was taken and strictly operated on according to the instructions of the kit (consistent with the Section 2.5.1 operation steps) to detect the levels of ATPase and Ca^2+^-ATPase in the kidney.

### 2.6. Non-Targeted Lipidomics Analysis

#### 2.6.1. Preparation of Lipid Samples

In a 5 mL centrifuge tube, 0.1 g of kidney samples were placed, and 600 L of pre-cooled distilled water was then added to vortex for 1 min, followed by 2.4 mL of pre-cooled methyl tert-butyl ether, vortex mixing for 1 min. Then, 720 L of pre-cooled methanol was added to continue voting for 1 min. The mixed samples were ultrasonicated in a low-temperature water bath for 20 min, then stood at room temperature for 30 min, centrifuged at 14,000 r·min^−1^ at 10 °C for 15 min, the upper organic phase was then absorbed, and dried with a nitrogen blow-dry concentrator to obtain a solid yellowish lipid powder. In the mass spectrometry analysis, 600 L of isopropanol/aacetonitrile/water solution with a volume ratio of 65:30:5 was added to the lipid sample. The complex solution was sufficiently vortexed, and the complex solution was centrifuged at 14,000 r·min^−1^ at 10 °C for 15 min. The supernatant was taken, passed through a 0.45 m organic filter membrane, transferred to a high-liquid tiny bottle liner, and analyzed by UPLC-Q-Orbitrap-HRMS.

QC samples: equal amounts of each group of samples are mixed into QC samples, and the QC samples are interspersed evenly throughout the samples to be tested.

#### 2.6.2. UPLC-Q-Orbitrap-HRMS Analysis

Non-targeted listings analysis of liver tissue UPLC-Q-Orbitrap-HRMS and DionexTMUltiMateTM3000 ultra-high performance liquid chromatography separation system were used to separate the lipid samples using an ESI ion source. During the whole experiment, the lipid samples were automatically injected into the sample tray at 10 °C, and the injection volume was 3 μL. Lipid separation was performed on a C18 column (100 mm × 2.1 mm, 1.7 μm) with a mobile phase flow rate of 300 μL/min and a column temperature of 45 °C. The mobile phase A was 60:40 (*v*/*v*) acetonitrile/water; mobile phase B was 10:90 (*v*/*v*) acetonitrile/isopropanol. Gradient elution procedure: 0–2 min, 30% B; 2–25 min, 100% B; 25–35 min, 30%. Table 3 displays the ion source settings and mass spectrometry scanning parameters.

### 2.7. Statistical Analysis

All the experimental data were analyzed by multiple analyses using the social science statistical package (SPSS 26 version) expressed as mean ± standard deviation (mean ± SD). A one-way analysis of variance (ANOVA) was used for inter-group differences, followed by a student’s test to assess significance. Statistically, significant results were signified as *p* < 0.05 and highly significant as *p* < 0.01 (compared with the blank control group: * *p* < 0.05, ** *p* < 0.01; compared with the model group: ^▲^ *p* < 0.05, ^▲▲^ *p* < 0.01).

## 3. Results

### 3.1. Effect of Ginseng Polysaccharide on Hexavalent Chromium-Induced Nephrotoxicity in Mice

#### 3.1.1. Effect of Ginseng Polysaccharide on the Physical Signs of Mice

**Figure 1:**
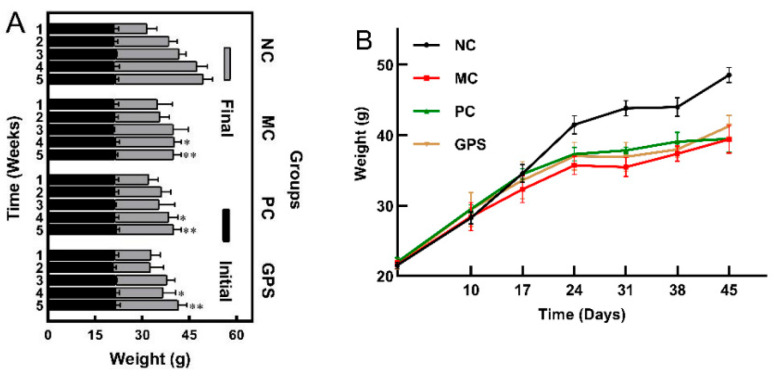
Time-effect evaluation for weight (**A**) Changes in the initial and final weights of mice in different batches and groups (an amount of 200 mice divided into five batches according to the increasing number of weeks of treatment). Compared with the NC group in this batch (weeks): * *p* < 0.05, ** *p* < 0.01. (**B**) Changes in the body weight of different groups of mice were compared throughout the experiment. Data are presented as mean ± SD.

**Figure 2:**
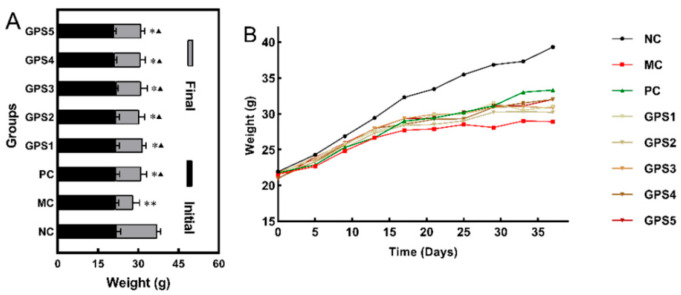
Dose−response evaluation for weight. (**A**) Changes in the initial and final weights of mice in different groups. Ginseng polysaccharide GPS1, 2, 3, 4, 5 (25, 50, 100, 200, 400 mg/kg/day). Compared with the NC group: * *p* < 0.05, ** *p* < 0.01. Compared with the MC group: ^▲^ *p* < 0.05. (**B**) Changes in body weight of different groups of mice were compared. Data are presented as mean ± SD.

The time-effect experiment is shown in Figure 1A and Figure 2A; there was no significant difference in the beginning body weight of mice in either group. There was no significant difference in the body weight among the four groups of mice three weeks before K_2_Cr_2_O_7_ exposure and drug treatment. After 4 weeks, the remaining three groups and the NC group showed significant differences (*p* < 0.01). As the experiment progressed, all K_2_Cr_2_O_7_-treated mice showed weight loss and inactivity. However, as compared to the MC group, the impacts of the PC and GPS groups were lower. Body weight was influenced by the timing of chromium intake in all groups except the NC group. The body weights of the MC group, PC group, and GPS group did not increase, even beginning to decrease the first week after administration of K_2_Cr_2_O_7_, as shown in Figure 1B. The results of the dose-effect experiment showed that, compared with the NC group and MC group (*p* < 0.05), the final body weights of the mice in the PC group and GPS group were significantly different at five different doses, as shown in Figure 2B. The body weight of the mice was affected by K_2_Cr_2_O_7_ exposure, and, compared to the other groups, the MC group was the most affected. Cr (VI) exposure combined with GPS treatment for 3–4 weeks at a dose of 100, 200, 400 mg·kg^−1^ alleviated the weight loss of the mice. No mice died during the whole experiment.

#### 3.1.2. Effect of Ginseng Polysaccharide on Kidney Index

Time-effect experimental results show that the kidney index of the MC group in five batches was significantly higher than that of the NC group (*p* < 0.01). After 3 weeks of treatment, the kidney index of the GPS group and the PC group was significantly different from that of the MC group (*p* < 0.05), and there was no significant difference with the NC group. The kidney index of mice in each group is shown in Table 4.

Dose–response experimental results show that the expression of the renal index in the MC group was significantly higher than that in the NC group (*p* < 0.01). The renal index in the PC group and GPS 3/4 (100/200 mg·kg^−1^) groups was significantly lower than that in the MC group (*p* < 0.01), which was similar to that in the NC group. The kidney index of mice in the GPS 1/2/5 (25/50/400 mg·kg^−1^) groups was significantly lower than that in the MC group (*p* < 0.05) but still significantly different from that in the NC group (*p* < 0.05). The kidney index of mice in each group is shown in Table 5.

#### 3.1.3. Effect of Ginseng Polysaccharide on Renal Function Biomarkers

**Figure 3:**
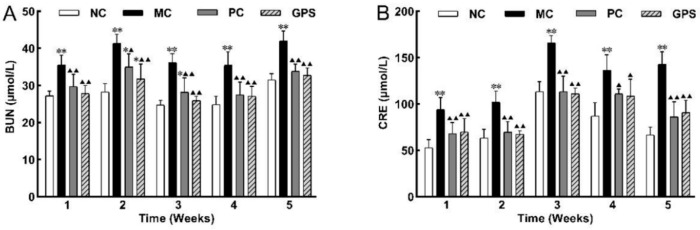
Time-effect of renal function evaluation. Changes in serum biochemical markers BUN (**A**) and CRE (**B**) values from 1 to 5 weeks of administration. Compared with the normal control group in this batch (weeks): * *p* < 0.05, ** *p* < 0.01. Compared with the model control group in this batch (weeks): ^▲^ *p* < 0.05, ^▲▲^ *p* < 0.01. Data are presented as mean ± SD.

After subcutaneous injection of K_2_Cr_2_O_7_, the expression levels of BUN and CRE (Figure 3) in the MC group increased significantly (*p* < 0.01) compared with those in the NC group in the five batches. After treatment with DMSA or GPS, the renal function of the mice was improved, and the values of BUN and CRE were significantly reduced (*p* < 0.01) compared with those in the MC group.

**Figure 4:**
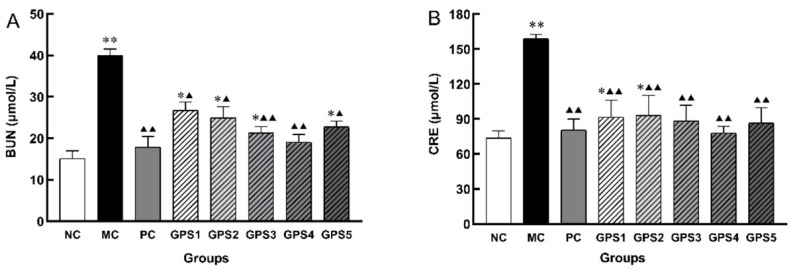
Dose–response of renal function evaluation. Changes in serum biochemical markers BUN (**A**) and CRE (**B**) at different doses. Ginseng polysaccharide GPS1, 2, 3, 4, 5 (25, 50, 100, 200, 400 mg/kg/day). Compared with the normal control group in this batch (weeks): * *p* < 0.05, ** *p* < 0.01. Compared with the model control group in this batch (weeks): ^▲^ *p* < 0.05, ^▲▲^ *p* < 0.01. Data are presented as mean ± SD.

After 39 d of K_2_Cr_2_O_7_ subcutaneous injection, the levels of BUN and CRE (Figure 4) were significantly increased in the MC group compared with the NC group (*p* < 0.01), and the downregulation of the levels of BUN and Cre in the middle-dose group was better than that in the high-dose and low-dose groups.

#### 3.1.4. Effect of Ginseng Polysaccharide on Renal Histopathology

HE staining is one of the most commonly used staining methods for paraffin sections. After hematoxylin staining, the nucleus and calcium mucus were blue. After proper treatment, the chromatin in the nucleus and the nucleic acid in the cytoplasm showed obvious blue−purple, while the cytoplasm and other components were decolorized. Plasma, red blood cells, muscle, connective tissue, and eosinophilic granules showed varying degrees of red or pink. Therefore, various tissue or cell components and the overall morphological and structural characteristics of the lesion can be displayed.

The results of the HE-stained paraffin profile showed that the kidneys of the NC mice were structurally normal and did not show histopathological deformation during the 1–5 weeks of drug administration. Regarding Cr (VI) after contamination, compared with the NC group, the kidney tissues of the mice in the MC group in 1–5 weeks showed edema and dilatation of tubular cells, detachment of tubular epithelial cells, and a minor amount of renal interstitial inflammatory cell infiltration, while the renal tissues showed focal lesions and the denaturation became serious along with the increase in the time of contamination.

After 1–4 weeks of DMSA treatment, the renal tubular cells, glomerular structural integrity, renal interstitial inflammatory cell infiltration, and pink granular degeneration were effectively improved in the five PC groups. DMSA showed an obvious effect 1–2 weeks after administration, and it still had a protective effect with the increase in Cr (VI) exposure time. However, after stopping administration and detoxification, the pathological changes in the kidney may relapse slightly. This shows that the use of DMSA does not provide a complete cure for Cr (VI)-induced toxicity.

**Figure 5:**
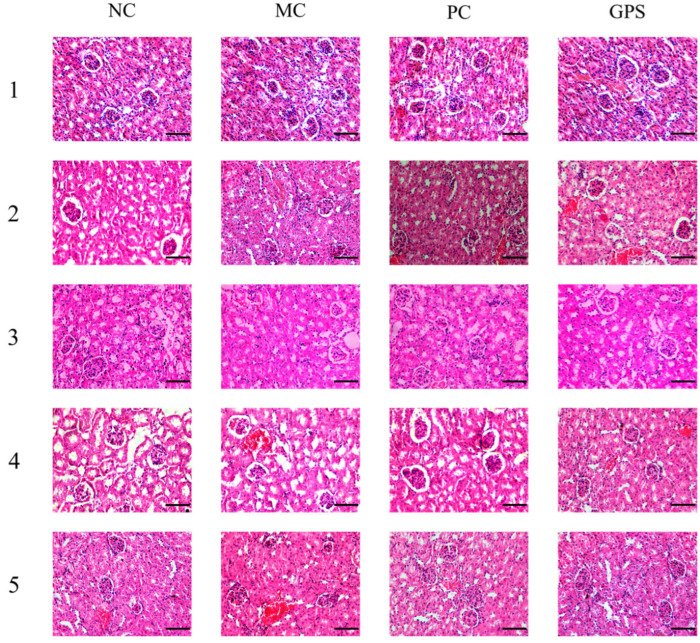
Pathological pictures of mouse kidney tissue in a time-effect experiment. NC: the blank control group; MC: the model control group; PC: DMSA treatment (70 mg·kg^−1^) group; GPS: Ginseng polysaccharide treatment (100 mg·kg^−1^) group. Scale bars: 50 μm. (HE stained, 400× magnification).

At 1–2 weeks after administration, the mice in the GPS administration group were still suffering from focal glomerular lesions and a slight edoema of renal tubular cells. At 3 weeks of administration, the overall morphology of the renal cells and glomerular structure in the GPS3 group returned to normal. When administration lasted for 4 weeks or after cessation of administration, the overall morphology of kidney tissues and cells in the GPS4 and GPS5 groups returned to normal; no further lesions were found or were and the state was similar to that of NC group. The kidney tissue sections of each group of mice are shown in Figure 5.

**Figure 6:**
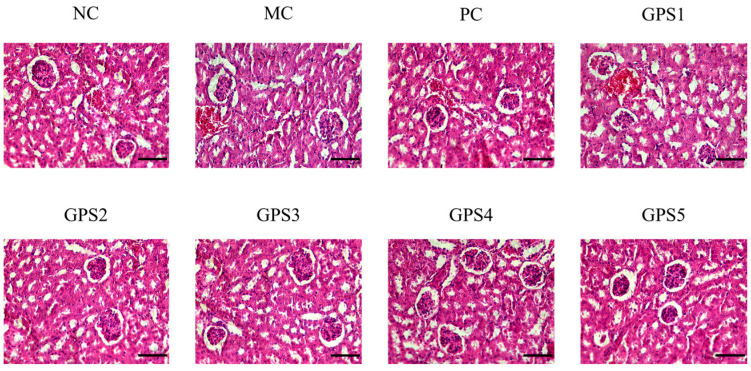
Pathological pictures of mouse kidney tissue in dose–response experiment. NC: the blank control group; MC: the model control group; PC: DMSA treatment (70 mg·kg^−1^) group; GPS 1/2/3/4/5: Ginseng polysaccharide treatment (25/50/100/200/400 mg·kg^−1^) group. Scale bars: 50 μm. (HE stained, 400× magnification).

By observing the pathological sections of kidney tissues, it can be seen that GPS administration can reduce Cr (VI)-induced nephrotoxicity in mice. After continuous administration of GPS for 3 weeks, the overall morphology of renal cells and the glomerular structure of mice were fundamentally restored. The morphology of the renal tissue and cells was virtually restored to that of the NC group in the medium dosage group. Figure 6 depicts kidney tissue slices from each group of mice.

### 3.2. Mechanism of Ginseng Polysaccharide against Hexavalent Chromium-Induced Nephrotoxicity in Mice

#### 3.2.1. Effect of Ginseng Polysaccharide on Oxidative Stress Index

**Figure 7:**
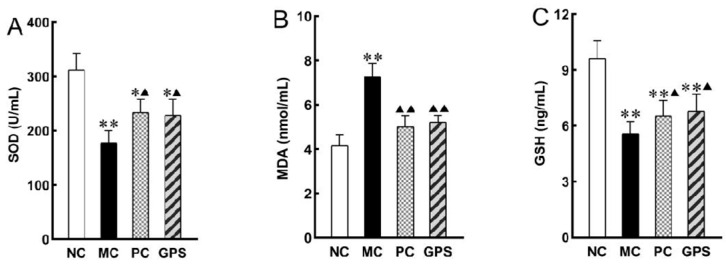
The effect of GPS pretreatment for three weeks on the oxidative-stress-induced K_2_Cr_2_O_7_ in the kidney. (**A**) The activity of SOD. (**B**) The content of MDA. (**C**) The level of GSH. Compared with the NC group: * *p* < 0.05, ** *p* < 0.01. Compared with the MC group: ^▲^ *p* < 0.05, ^▲▲^ *p* < 0.01. Data are presented as mean ± SD.

The results showed that the accumulation of Cr (VI) in the body caused damage to the kidney functions of mice. By inducing oxidative stress in cells, SOD and GSH, as antioxidants, scavenged ROS produced in the physiological metabolism of mice, resulting in consumption and a significant decrease in SOD (Figure 7A) and GSH (Figure 7C) levels (*p* < 0.01). The content of MDA (Figure 7B) increased significantly (*p* < 0.01), indicating that the accumulation of Cr (VI) led to membrane lipid peroxidation in the body and that the degree of oxidative damage in the kidney tissue was enhanced, resulting in damage to kidney cells or cell membranes. This was improved with DMSA or GPS treatment.

#### 3.2.2. Effect of Ginseng Polysaccharide on Apoptosis Index

**Figure 8:**
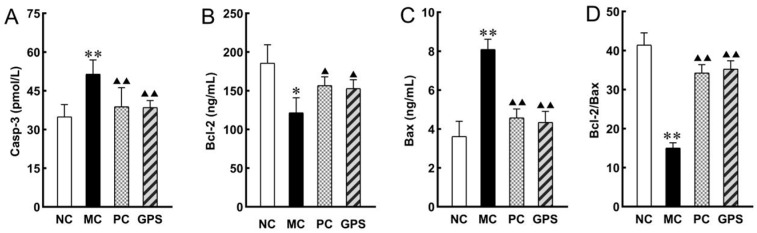
The effect of GPS pretreatment for three weeks on K_2_Cr_2_O_7_-induced kidney cell apoptosis. (**A**) The expression level of Casp-3. (**B**) The expression level of Bcl-2. (**C**) The expression level of Bax. (**D**) Bcl-2/Bax ratio. Compared with the NC group: * *p* < 0.05, ** *p* < 0.01. Compared with the MC group: ^▲^ *p* < 0.05, ^▲▲^ *p* < 0.01. Data are presented as mean ± SD.

Casp-3 activity was greater in the MC group than in the NC group (*p* < 0.01) (Figure 8A). In the K_2_Cr_2_O_7_-exposed group, Bcl-2 expression was dramatically downregulated (Figure 8B), and Bax expression was significantly raised (*p* < 0.05) (Figure 8C). The Bcl-2/Bax ratio was reduced in the MC group compared to the NC group (*p* < 0.01) (Figure 8D). The ratio of Bcl-2/Bax was significantly increased after DMSA and GPS treatment (*p* < 0.01). There was confirmation that GPS or DMSA may alleviate Cr (VI) exposure-induced apoptosis in renal cells by inhibiting the upregulation of Casp-3 apoptotic protein as well as Bax pro-apoptotic protein and promoting the expression of Bcl-2 genes.

#### 3.2.3. Effect of Ginseng Polysaccharide on Cell Membrane Damage Index

**Figure 9:**
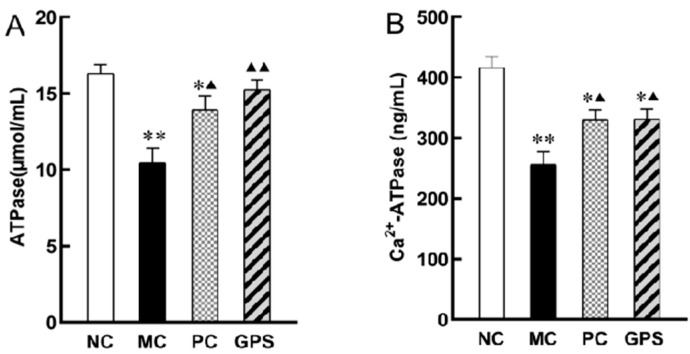
The effect of GPS pretreatment for three weeks on K_2_Cr_2_O_7_-induced kidney cell membrane damage. (**A**) The expression level of ATPase. (**B**) The expression level of Ca^2+^-ATPase. Compared with the NC group: * *p* < 0.05, ** *p* < 0.01. Compared with the MC group: ^▲^ *p* < 0.05, ^▲▲^ *p* < 0.01. Data are presented as mean ± SD.

Cr (VI) poisoning resulted in a significant downregulation of ATPase expression in mouse kidney tissue (*p* < 0.01). After 3 weeks of treatment, the level of ATPase in the PC group was significantly upregulated (*p* < 0.05), while that in the GPS4 group recovered to a similar level to the NC group (*p* < 0.01) (Figure 9A). The activity of Ca^2+^-ATPase in the K_2_Cr_2_O_7_ exposure group was significantly lower than that in the NC group (*p* < 0.01). After 3 weeks of GPS or DMSA treatment, renal Ca^2+^-ATPase activity was significantly higher than that in the MC group (*p* < 0.05). Compared with the NC group, the treatment improved the Ca^2+^-ATPase activity of the mice but still did not completely return to normal (*p* < 0.05) (Figure 9B).

### 3.3. Multivariate Analysis and Structural Identification of Differential Lipids

**Figure 10:**
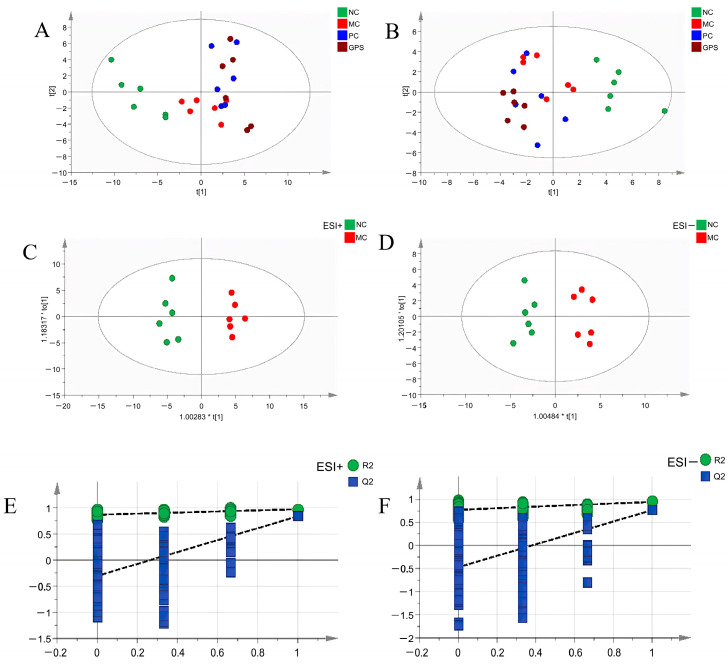
The PCA scores of the ion mode (**A**) and the negative ion mode (**B**) of serum lipid analysis in each experimental group. The OPLS-DA scores of the positive mode (**C**) and the negative mode (**D**) of serum lipid analysis in each experimental group, as well as the cross-validation of the positive mode (**E**) and the negative mode (**F**).

The normalized data of NC, MC, PC, and GPS were imported into Simca14.1 to establish an unsupervised PCA model and a supervised OPLS-DA model for multivariate statistical analysis. Firstly, PCA was used for unsupervised data analysis to observe the intra-group clustering, inter-group distribution, and the outlier samples of each group. Subsequently, the outlier samples were removed to obtain the PCA score map of the data collected in the positive and negative ion modes, as shown in Figure 10A,B. The results indicate that there were inherent differences in the overall lipid metabolism levels between the NC group and the MC group, while the overall lipid metabolism levels of mice after intragastric administration (DMSA and GPS groups) had a tendency to shift to normal levels, indicating that DMSA and GPS had the effect of alleviating the overall lipid metabolism abnormalities caused by Cr (VI).

An OPLS-DA model was created in five groups to display lipid alterations and enable the screening of differential lipids (Figure 10C,D). In positive ion mode, R2X = 0.462, R2Y = 0.971, Q2 = 83.6%; in negative ion mode, R2X = 0.877, R2Y = 1, Q2 = 90.2%, indicating that the fitting and prediction abilities are satisfactory. The difference between R2Y and Q2 in both modes is less than 0.3, and Q2 is greater than 90%. In general, when Q2 is greater than 50%, the fitting degree and prediction ability of the model are favorable. It can be seen that the fitting degree and prediction ability of the model in positive and negative ion modes are excellent. The OPLS-DA model over-fitting phenomenon that may occur due to over-magnification of the difference between the NC group and the MC group was prevented after 200 permutation verifications. The results of 200 permutations show that the Y-axis intercept of the Q2 regression line is −0.3036 in positive ion mode (Figure 10E). In the negative ion mode, the Y-axis intercept of the Q2 regression line is −0.4686 (Figure 10F), which proves that the OPLS-DA model does not produce over-fitting. The aforementioned assessment and verification of model parameters demonstrate the statistical significance of the built models. Based on this, the OPLS-DA score map showed a clear separation trend between the NC group and the MC group, which additionally proved that Cr (VI) poisoning caused differences in overall lipid metabolism in kidney tissue.

The data between the NC group and the MC group were compared and analyzed. Lipids that met the conditions of VIP ≥ 1.0, FC ≥ 1.5 or FC ≤ 0.67 and *p* ≤ 0.05 were used as differential lipid metabolites. By comparing the difference variables of MS/MS spectra with the standard MS/MS spectra of candidate lipids in the HMDB database, 17 differential lipids were accurately identified. These included glycerophospholipids, pregnenolone lipids, fatty acids, sphingolipids, and glycerolipids. The detailed results are shown in Table 6.

**Figure 11:**
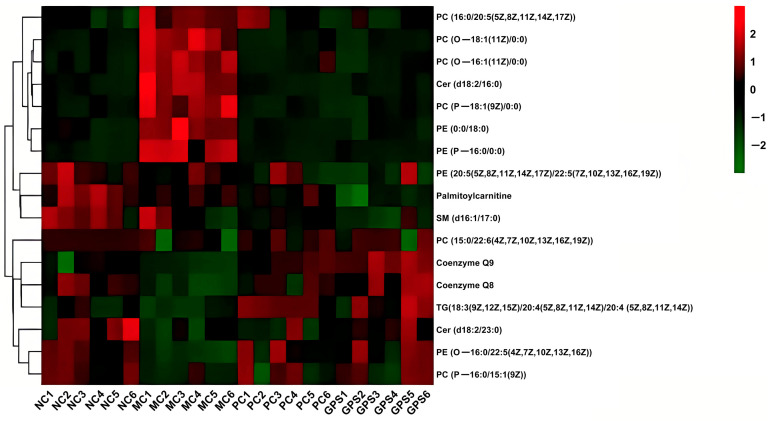
Differential lipid heatmap.

A total of 17 differential lipid metabolism levels between the NC, MC, PC, and GPS groups were analyzed by a heat map. Red indicated that lipid metabolites were upregulated, and green indicated that they were downregulated. As shown in Figure 11, overall, the expression of 17 differential lipids in the MC group and NC group showed significant differences. Most lipid metabolites were similarly expressed among NC, PC and GPS groups. This suggests that the MC group mice exposed to Cr (VI) had affected lipid expression levels in their kidney tissues, significantly modulating PC and PE expression levels. The findings explain that GPS against Cr (VI)-induced nephrotoxicity may genuinely protect the organism by modulating the expression of differential lipids.

## 4. Discussion

Chromium, as a common heavy metal, is widely distributed in the environment and poses a serious threat to human health. Chromium toxicity leads to immunosuppression of the body, which makes it more susceptible to disease. Numerous studies have shown that hexavalent chromium is not easily eliminated from the environment, is easily bioconcentrated through the food chain, and can strongly irritate the skin and mucous membranes, producing organ toxicity when it enters the body, with one of the most affected organs being the kidney. A large number of studies have proved that plant polysaccharides can effectively alleviate many forms of kidney injury, which makes polysaccharides widely used in the treatment of kidney diseases. Among them, ginseng polysaccharides have been proven to have pharmacological effects in terms of antioxidants, slowing down depression, preventing Alzheimer’s disease, ameliorating atherosclerosis, improving osteoblast survival, and inhibiting tumor cell growth. Research shows that ginseng polysaccharides can also prevent gastric inflammation and oxidative stress in ethanol-ethical rats by modulating nuclear transcription factor signaling pathways [25].

Studies have shown that the growth rate of mice after hexavalent chromium poisoning has slowed significantly, and the weight of mice has also decreased significantly. After K_2_Cr_2_O_7_-treated mice were supplemented with ginseng polysaccharide, their growth rate and average daily feed intake significantly increased, and their body weight also increased. Chromium, as a common heavy metal, is widely distributed in the environment and severely threatens human health. Chromium poisoning can lead to immunosuppression and make the body more susceptible to disease. Numerous domestic studies have shown that ginseng can play a beneficial role in the central nervous system and metabolic system, and it can also be used to treat infectious and oncological diseases. This suggests that our ginseng polysaccharides may be able to enhance immunity, alleviate liver dysfunction, and enhance intestinal barrier function, thereby promoting growth in hexavalent chromium-intoxicated mice.

A large number of studies have shown that hexavalent chromium is not easy to eliminate in the environment and is susceptible to bio-enrichment through the food chain. After entering the body, it can heavily stimulate the skin and mucosa, producing organ toxicity. The kidney is one of the most affected organs. The findings of this experiment revealed that the renal index of the model group rose dramatically when compared to the blank control group and that ginseng polysaccharide administration may lower the kidney index of hexavalent-chromium-poisoned mice. The observation of renal histopathology sections also confirmed that hexavalent chromium poisoning caused renal injury. With the increase in Cr (VI) exposure time, the renal histopathological sections of mice in the MC group gradually showed pathological phenomena such as renal tubular cell edema, renal tubular epithelial cell injury, and inflammatory cell infiltration in the intercellular substance. After treatment with GPS or DMSA, the recovery was improved, but the recovery degree of the evaluation index was not linear with the time and dose of GPS administration. Numerous investigations have shown that plant polysaccharides can effectively alleviate multiple forms of kidney injury, which makes polysaccharides widely used in the treatment of kidney diseases. Ginseng polysaccharide has been proven to have pharmacological effects on anti-oxidation, depression alleviation, prevention and treatment of senile dementia, improvement of atherosclerosis, improvement of osteoblast survival rate, and inhibition of tumor cell growth. 

BUN and Cre levels are commonly used to reflect renal functional status. Both are nitrogenous end products of metabolism; BUN is a major metabolite of dietary and tissue protein turnover, and Cre is a product of muscle creatine breakdown. Ginsenoside 20 (R)-Rg3 effectively inhibits the elevation of BUN levels in mouse serum and ameliorates d-galactose-induced hepatic and renal injuries in senescent mice by suppressing oxidative stress and inhibiting PI3K/Rg3 protein kinase activity [26]. Research shows that ginseng polysaccharides can improve renal function by gradually reversing the expression of α-SMA protein in the renal cortex of diabetic mice and ultimately delaying the onset and progression of renal fibrosis [27]. The present study showed that all groups of mice exposed to Cr (VI) exhibited elevated BUN and CRE, and the BUN and CRE levels exhibited after ginseng polysaccharide administration and treatment were significantly ameliorated and restored, suggesting that ginseng polysaccharides may block the progression of renal injury.

Based on the above conclusions, the protective mechanism of ginseng polysaccharides against the nephrotoxicity caused by hexavalent chromium was investigated. MDA is the final decomposition product of membrane lipid peroxidation, which often occurs when the body ages or suffers from injuries, and the MDA content can clearly reflect the degree of the body’s injuries. The release and accumulation of MDA can result in the loss of function of proteins and nucleic acids, causing damage to cells and cell membranes. Research shows that MDA reflects the intensity of the lipid peroxidation rate in the body and indirectly reflects the degree of peroxidative damage to tissues [28]; therefore, MDA content is a commonly used indicator in anti-heavy metal toxicity studies. The intracellular reduction of Cr (VI) to Cr (III) induces the formation of oxygen radicals, ROS, and intermediates such as Cr (V) and Cr (IV), which are the key to the oxidative damage effects of Cr (VI), such as cellular lipid peroxidation, biofilm disruption, and damage to biomolecules [29,30]. The production of reactive oxygen species exceeds the ability of the antioxidant system to protect cells from oxidizing molecules, and oxidative stress occurs as a result. SOD, an antioxidant metalloenzyme present in living organisms, catalyzes the disproportionation of superoxide anion radicals to produce oxygen and hydrogen peroxide, which provides the first line of defense against free radicals, while GSH scavenges the oxygen and hydrogen peroxide produced [31]. According to Jin’s study, the protective mechanism of ginseng is that it can minimize renal injury, stress, inflammatory response, epithelial-mesenchymal transition, and fibrosis by inhibiting oxidative stress, inflammatory response, and epithelial cell damage [32]. The results of the present experiment confirmed the above conclusion that the levels of SOD and GSH were significantly decreased in the model group, while the level of MDA was significantly increased in the model group as compared to the blank control group. This indicated that hexavalent chromium induced oxidative stress and lipid peroxidation in mice. The ginseng polysaccharide administration group significantly increased the levels of GSH and SOD and decreased the levels of MDA in hexavalent chromium-intoxicated mice compared to the model group.

Ginseng polysaccharides and ginsenoside Rg1 have been reported to ameliorate oxidative stress injuries in diabetic rats [33]. Renal apoptosis is associated with the development of oxidative stress and inflammation [34]. Therefore, apoptosis markers were tested in this experiment. The results of this experiment indicated that the expression of Casp-3 and Bax was significantly higher in the model group, while the expression of Bcl-2 was significantly lower compared to the blank control group. The Bcl-2 gene is an oncogene that significantly inhibits apoptosis [35]. Bax belongs to the Bcl-2 family and is one of the extremely important pro-apoptotic genes, encoding a Bax protein that forms a heterodimer with Bcl-2 and exerts a blocking effect on Bcl-2. The proportionality between the two Bcl-2/Bax proteins is a key factor in determining the inhibition of apoptosis [36]. Caspases play a key role in apoptosis. Casp-3 is a protease that is the predominant terminal shear enzyme in apoptosis and is essential for this process, activating all stages of cell death in a non-invasive manner [37]. As apoptosis proceeds, Casp-3 transmits apoptotic signals and activates preceding caspase proteins. Therefore, a deeper understanding of Casp-3 in apoptosis will probably reveal the mechanisms by which damage such as this occurs and help in the search for therapeutic avenues. Research shows that showed that Cr (VI)-induced mitochondrial damage is the mechanism of its hepatotoxic effects, suggesting that this process is related to the induction of an inflammatory response and oxidative stress [38]. Strikingly, the results of this experiment showed that ginseng polysaccharides significantly reduced the concentrations of Casp-3 enzyme and Bax protein, thus increasing the concentration of Bcl-2 protein in the kidney tissues of hexavalent chromium-poisoned mice. Wang’s research showed that ginseng has an anticancer effect in vitro in the treatment of prostate cancer. The mechanism may induce apoptosis through the mitochondrial pathway, production of ROS, and inhibition of cell proliferation [39]. Zhu’s research showed that ginseng extract reduced the number of glial fibrillary acidic protein-immunoreactive cells, also reducing apoptosis by upregulating Bcl-2 and downregulating Bax protein expression [40]. Ginseng polysaccharides could protect mitochondria by inhibiting mitochondrial swelling and improving energy metabolism [41]. These results suggest that ginseng can protect injury of the kidney from Cr (VI)-induced apoptosis by upregulating Bcl-2 expression, downregulating Bax and Casp-3 expression, and improving mitochondrial dysfunction. Previous studies have shown that polysaccharides play a beneficial role by regulating the immune function of macrophages [42]. Ginseng leaf polysaccharide can affect the activity of macrophages and play an anti-tumor role [43]. At the same time, the infiltration and polarization of macrophages play a vital role in the process of renal injury [44,45]. Jiao et al. According to Jiao’s study, M2macrophages mediate renal fibrosis through the transition of monocyte/macrophage to myofibroblast [46,47]. However, no studies have shown that ginseng polysaccharides can alleviate hexavalent chromium-induced renal injury by regulating macrophages. It is suggested that we can carry out research in this area in the future to further explore the mechanism of ginseng polysaccharide in the treatment of renal injury caused by hexavalent chromium.

On the basis of the previous efficacy evaluation and the exploration of the traditional target mechanisms, the active mechanism of GPS against Cr (VI)-induced nephrotoxicity was investigated at a deeper level than the non-targeted lipidomic level. Biological membranes play a key role in cellular life, acting as permeability barriers and constituting privileged sites of communication between the inside and outside of the cell. The main structural element of biological membranes is the phospholipid bilayer, and glycerophospholipids, as a type of phospholipid, are its main component [48]. The bilayer provides a support matrix for many enzymatic reactions, is involved in signal transduction, provides precursors for signaling processes and macromolecular synthesis, and is susceptible to oxidative damage caused by reactive oxygen species (ROS) [49]. Intracellular phospholipids, lysophosphatidylcholine, and free fatty acids can be transformed into each other through the “lands cycle”, and phospholipase A (PLA) is the key enzyme controlling phospholipid metabolism in the “lands cycle” [50]. The three most important substances in glycerophospholipids are phosphatidylcholine (PC), phosphatidylethanolamine (PE), and phosphatidylinositol (PI) [51]. In vivo, PC, PE, and PI can be hydrolyzed by PLA into single-chain lysophospholipids and free fatty acids [52]. PC and PE play a role in maintaining lipid metabolism homeostasis in organisms, and changes in their content will directly affect the fluidity and permeability of cell membranes, thus affecting the intracellular transport of substances and the localization of membrane proteins [53]. In conclusion, GPS can effectively exert a renoprotective effect and improve lipid metabolism disorders caused by Cr (Ⅵ) exposure by regulating the glycerophospholipid metabolism signaling pathway in the kidney.

Our study also has some limitations. First, no specific mechanism or signaling molecule has been identified for the renoprotective mechanism of GPS, and the mechanism of action of GPS against Cr (VI)-induced nephrotoxicity should be exhaustively explained in future studies. Secondly, DMSA is a heavy metal detoxifier, and different positive drugs (e.g., nutrients, vitamins, etc.) were added to strengthen the comparison, to observe and study the differences and similarities of their pharmacodynamic effects, and to improve the evaluation of the advantages and shortcomings of GPS in exerting the nephrotoxicity of Cr (VI).

## 5. Conclusions

**Figure 12:**
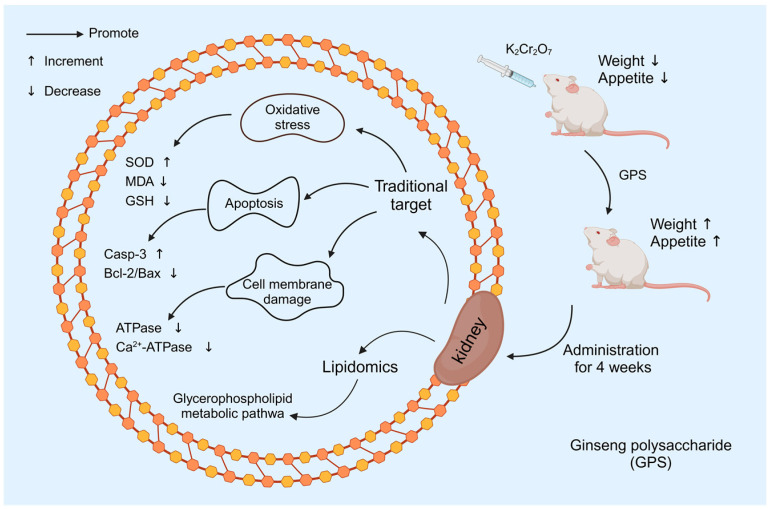
Mechanism of hexavalent chromium-induced renal injury in mice.

In summary, oral administration of GPS reduced renal injury in ICR mice; it also alleviated oxidative stress and inhibited renal cell apoptosis, thereby attenuating the nephrotoxicity produced by Cr (VI) exposure to the organism. These results indicate that CPS is a therapeutically effective plant polysaccharide and suggest that GPS may be used as a nutritional and adjunctive medicine based on ginseng for nephrotoxicity. The mechanism of hexavalent chromium-induced renal injury in mice is shown in Figure 12.

## Figures and Tables

**Table 1:** Schematic table of time-effect experimental scheme.

Group	Administration time (Week)											
	0	1		2		3		4		5		
	Experiment time (Day)											
	1–10	11–16	17	11–23	24	11–30	31	11–37	38	11–37	37–44	45
NC	○	○○	Sacrifice (Renal Function)	○○	Sacrifice (Renal Function)	○○	Sacrifice (Renal Function)	○○	Sacrifice (Renal Function)	○	Stop/Continuous treatment	Sacrifice (Renal Function)
MC	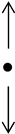	●○		●○		●○		●○		●		
PC		●□		●□		●□		●□		●		
GPS		●■		●■		●■		●■		●		

All administered by gavage (0.1 mL/10 g/day). ○: saline ●: potassium dichromate (K_2_Cr_2_O_7_ 50 mg/kg/day). □: positive drug dimercaptosuccinic acid (DMSA 70 mg/kg/day). ■: ginseng polysaccharide (GPS 100 mg/kg/day).

**Table 2:** Schematic table of dose-effect experimental scheme.

Group	Administration Time (Days)		
	1–10	11–30	31
NC	○	○○	Sacrifice (Renal Function)
MC	●	●○	
PC		●□	
GPS1		●■	
GPS2		●■	
GPS3		●■	
GPS4		●■	
GPS5		●■	

■: ginseng polysaccharide GPS1, 2, 3, 4, 5 (25, 50, 100, 200, 400 mg/kg/day). The other doses are the same as in Table 1.

**Table 3:** Ion source parameters and mass spectrometry scanning parameters.

Condition	Positive Ion Mode	Negative Ion Mode
Heating temperature (°C)	300	300
Sheath gas velocity (arb)	45	45
Auxiliary gas velocity (arb)	15	15
Purge gas flow rate (kv)	1	1
Spray voltage (°C)	3	2.5
Ion transport tube temperature (°C)	350	350
S-lens voltage (%)	50	50
Mass scanning range of primary mass spectrometry (Da)	200–1800	250–1800

**Table 4:** Changes in kidney index in mice studied by a time-effect experiment (mean ± SD, *n* = 7, %).

Group	1 Week	2 Weeks	3 Weeks	4 Weeks	5 Weeks
NC	0.53 ± 0.055	0.540 ± 0.015	0.548 ± 0.025	0.513 ± 0.034	0.516±0.016
MC	0.62 ± 0.048 *	0.600 ± 0.022 **	0.603 ± 0.055 *	0.576 ± 0.050 *	0.581 ± 0.061 *
PC	0.58 ± 0.050	0.554 ± 0.025 ^▲^	0.553 ± 0.031 ^▲^	0.519 ± 0.072 ^▲^	0.541 ± 0.041
GPS	0.60 ± 0.043 *	0.569 ± 0.037 ^▲^	0.553 ± 0.021 ^▲^	0.530 ± 0.028	0.521 ± 0.041 ^▲^

* *p* < 0.05, ** *p* < 0.01 vs. the normal control group (NC). ^▲^ *p* < 0.05 vs. the model control group (MC).

**Table 5:** Changes in kidney index in mice studied by a dose–response experiment (mean ± SD, *n* = 7%).

Group	Renal Index	Group	Renal Index	Group	Renal Index
NC	0.58 ± 0.037	MC	0.63 ± 0.055 **	PC	0.59 ± 0.051 ^▲▲^
GPS 1	0.60 ± 0.070 *^▲^	GPS 2	0.61 ± 0.057 *^▲^	GPS 3	0.58 ± 0.046 ^▲▲^
GPS 4	0.59 ± 0.051 ^▲▲^	GPS 5	0.60 ± 0.0370 *^▲^		

Ginseng polysaccharide GPS1, 2, 3, 4, 5 (25, 50, 100, 200, 400 mg/kg/day). * *p* < 0.05, ** *p* < 0.01 vs. the normal control group (NC). ^▲^ *p* < 0.05, ^▲▲^ *p* < 0.01 vs. The model control group (MC).

**Table 6:** Screening and identification information of differential metabolites.

No.	Name	EIS Mode	Measured m/z	Retention Time (min)	Category	Mass Accuracy (ppm)	MS/MS Fragments	MC	GPS
1	PE(0:0/18:0)	−	481.3156	3.559	Glycerophospholipids	−2	78.9576; 283.2636	↑	↓
2	Coenzyme Q9	+	794.6198	17.446	Pregnenolone lipids	−1	81.0703; 197.0802	↓	↑
3	PC (15:0/22:6(4Z,7Z,10Z,13Z,16Z,19Z))	−	791.5456	13.411	Glycerophospholipids	−1	78.9575; 283.2654; 327.2320	↓	↑
4	PE (O- 16:0/22:5(4Z,7Z,10Z,13Z,16Z))	+	751.5505	14.227	Glycerophospholipids	−1	361.2726; 392.2928	↓	↑
5	Cer (d18:2/16:0)	−	535.4953	12.486	sphingolipid	−2	280.2637; 534.4876	↑	↓
6	PC (O- 18:1(11Z)/0:0)	+	504.3477	2.629	Glycerophospholipids	−2	86.0967; 104.1071	↑	↓
7	PE (P- 16:0/0:0)	−	437.2894	2.590	Glycerophospholipids	−2	78.9576; 140.0104; 196.0369; 239.2372; 436.2820	↓	↑
8	Cer (d18:2/23:0)	−	633.6046	16.115	sphingolipid	−2	280.2637; 534.4876	↓	↑
9	Palmitoylcarnitine	+	399.3334	1.943	fatty acids	−3	393.2962	↓	↑
10	Coenzyme Q8	+	726.5566	16.373	Pregnenolone lipids	−2	86.0968; 664.4657; 723.5400	↓	↑
11	PC (O- 16:1(11Z)/0:0)	+	479.3362	1.706	Glycerophospholipids	−2	415.2201; 433.2311	↑	↓
12	PC (16:0/20:5(5Z,8Z,11Z,14Z,17Z))	−	779.5438	10.39	Glycerophospholipids	−3	78.9576; 303.2322	↑	↓
13	PC (P- 18:1(9Z)/0:0)	+	505.3516	1.889	Glycerophospholipids	−3	86.0968; 104.1072	↑	↓
14	PE (20:5(5Z,8Z,11Z,14Z,17Z)/22:5 (7Z,10Z,13Z,16Z,19Z))	+	811.5120	11.019	Glycerophospholipids	−3	812.5469	↑	↓
15	SM (d16:1/17:0)	+	688.5497	10.968	sphingolipid	−3	184.0728	↓	↑
16	PC (P- 16:0/15:1(9Z))	+	701.5345	14.205	Glycerophospholipids	−2	184.0727	↓	↑
17	TG(18:3(9Z,12Z,15Z)/20:4(5Z,8Z,11Z,1 4Z)/20:4 (5Z,8Z,11Z,14Z))	+	924.7156	18.717	glycerolipid	−5	601.5157	↓	↑

The up arrows (or down arrows) represented the relative upregulation (or downregulation) of the differential lipids.

## Data Availability

Data is contained within this article.
